# Risk Stratification in Differentiated Thyroid Cancer: An Ongoing Process

**DOI:** 10.5041/RMMJ.10230

**Published:** 2016-01-28

**Authors:** Gal Omry-Orbach

**Affiliations:** Department of Endocrinology, Diabetes and Metabolism, Virginia Mason Medical Center, Seattle, WA, USA

**Keywords:** Cancer, risk stratification, thyroid

## Abstract

Thyroid cancer is an increasingly common malignancy, with a rapidly rising prevalence worldwide. The social and economic ramifications of the increase in thyroid cancer are multiple. Though mortality from thyroid cancer is low, and most patients will do well, the risk of recurrence is not insignificant, up to 30%. Therefore, it is important to accurately identify those patients who are more or less likely to be burdened by their disease over years and tailor their treatment plan accordingly. The goal of risk stratification is to do just that. The risk stratification process generally starts postoperatively with histopathologic staging, based on the AJCC/UICC staging system as well as others designed to predict mortality. These do not, however, accurately assess the risk of recurrence/persistence. Patients initially considered to be at high risk may ultimately do very well yet be burdened by frequent unnecessary monitoring. Conversely, patients initially thought to be low risk, may not respond to their initial treatment as expected and, if left unmonitored, may have higher morbidity. The concept of risk-adaptive management has been adopted, with an understanding that risk stratification for differentiated thyroid cancer is dynamic and ongoing. A multitude of variables not included in AJCC/UICC staging are used initially to classify patients as low, intermediate, or high risk for recurrence. Over the course of time, a response-to-therapy variable is incorporated, and patients essentially undergo continuous risk stratification. Additional tools such as biochemical markers, genetic mutations, and molecular markers have been added to this complex risk stratification process such that this is essentially a continuum of risk. In recent years, additional considerations have been discussed with a suggestion of pre-operative risk stratification based on certain clinical and/or biologic characteristics. With the increasing prevalence of thyroid cancer but stable mortality, this risk stratification may identify those in whom the risk of conventional surgical treatment may outweigh the benefit. This review aims to outline the process of risk stratification and highlight the important concepts that are involved and those that are continuously evolving.

## INTRODUCTION

Thyroid cancer has become the most common endocrine malignancy, with an increasing incidence over the past 20 years. It is estimated that 1.1% of men and women will be diagnosed with thyroid cancer in their lifetime. In the United States, thyroid cancer is expected to account for 3.8% of new cancer diagnoses. Prognosis, however, remains excellent, with a 97.9% 5-year survival.^1^ In Israel, in 2012 (the most recent year for which there is current published information), thyroid cancer accounted for 3.4% of new cancer diagnoses.^2^ Despite the excellent outcome, there are some in whom the course is not indolent. Up to 30% of patients diagnosed with thyroid cancer may experience a clinically significant recurrence over their lifetime. These people require more aggressive monitoring and treatments. The goal of risk stratification is accurately to tailor a treatment and monitoring plan to minimize risk and maximize benefit.

## POST-OPERATIVE STAGING—RISK STRATIFICATION FOR MORTALITY

Following initial surgery, application of American Joint Committee on Cancer/Union for International Cancer Control (AJCC/UICC) staging^3^ is a universally accepted and standard tool for classification of thyroid cancer. This staging system classifies patients based on age and histopathologic findings and is essentially the first systematic risk stratification iteration that the patient will undergo once the diagnosis of thyroid cancer is confirmed. The AJCC/UICC staging system is designed to be used to predict disease-specific mortality; however, it is limited in its ability to account for variables that can affect long-term prognosis. Over the years, a number of additional staging systems have been proposed that encompass other variables thought to be predictors of prognosis, including but not limited to EORTC,^4^ AGES,^5^ AMES,^6^ MACES,^7^ and MSK.^8^ Though one method has not definitively been shown to be superior, a number of studies have suggested that MACES may be the most appropriate predictor of survival in differentiated thyroid cancer, both papillary^9^ and follicular thyroid cancer.^10^,^11^ The AJCC/UICC staging system and these noted above are unable sufficiently to assess the risk of recurrence and therefore do not accurately direct the decision-making process with regard to nature and frequency of treatment and monitoring.

There are several factors not included in AJCC that may affect risk of recurrence/persistence. Histologic subtypes of papillary thyroid cancer (PTC), such as tall cell variant, diffuse sclerosing variant, columnar cell variant, hobnail variant, as well as widely invasive follicular thyroid cancer (FTC), or poorly differentiated thyroid cancer, are all are thought to have a more aggressive nature. Conversely, other variants such as encapsulated follicular variant PTC or minimally invasive FTC are thought to be more favorable.^12^–^14^

When lymph nodes are involved at the time of the original treatment, the size of the metastatic lymph node and/or the presence of extra-capsular spread can affect the risk of disease recurrence/persistence and distant metastatic disease.^15^,^16^

Vascular invasion too has prognostic importance, as it can be associated with an increased risk of long-term disease.^17^,^18^

Patients with encapsulated follicular variant papillary thyroid cancer frequently have the RAS mutations,^19^ and their disease is often indolent.^20^ In contrast, non-encapsulated follicular variants often harbor BRAF V600E mutations and can follow a more aggressive path.^16^,^21^

Further, the AJCC staging system does not account for clinical risk factors that may affect outcome such as degree and extension beyond thyroid tissue, details of any remaining gross disease or completeness of resection, or vocal cord paralysis preoperatively.

As such, though of certain value in the initial risk stratification and useful in guiding the treating clinician in terms of initial post-surgical treatment, these staging systems are limited in the post-initial-treatment course and fail precisely to dictate who will require more aggressive treatment and monitoring versus who, due to very low risk of recurrent or persistent disease, can avoid these. Because of the significant difference in the post-initial-treatment course in those who will remain disease-free versus those who will not, a concept of risk-adaptive management of thyroid cancer has been proposed.^22^,^23^

## POST INITIAL THERAPY—INITIAL RISK STRATIFICATION FOR PERSISTENCE/RECURRENCE

Paradigms have shifted over the course of the past 20 years. Formerly, emphasis was placed on early disease detection with the thought that detection of small-volume disease will lead to better clinical outcomes. That said, the majority of patients will not have recurrence.

It is important to consider the ramifications of unnecessary intense monitoring, including the increased financial burden on patients and the health care system, as well as the psychosocial impact of aggressive testing for disease that will have little clinical impact over time. Conversely, patients initially thought to be at low risk for recurrence based on their original classification may have an incomplete response to therapy that without appropriate surveillance may be left undiagnosed and thus not appropriately treated. Without inclusion of a response-to-therapy variable, patients originally classified as low risk who do not respond as expected to initial therapy may be subjected to follow-up testing which is less than optimal. Alternatively, moderate-to-high-risk patients who demonstrate a complete response to initial therapy may still be considered at high risk for recurrence and thus encumbered by follow-up that is more intensive than necessary. Thus, risk stratification should be a dynamic process which may change over time based on follow-up testing and additional information obtained.

Approximately 10 years ago, various authoritative guidelines for the management of thyroid cancer had started to include an estimated risk of disease-specific death and/or recurrence in the recommendations for initial treatment and follow-up.^24^,^25^

In 2009, the revised guidelines for treatment of thyroid cancer from the American Thyroid Association (ATA)^26^ incorporated a risk stratification system to predict risk of disease recurrence. With this system, patients were categorized as low risk, intermediate risk, and high risk. Variables include presence/absence of local and distant metastatic disease, degree of gross tumor resection, loco-regional tumor invasion, histology (more or less aggressive, as discussed above), vascular invasion, as well as extent of uptake seen on a post-therapy whole-body scan if I-131 was administered.

## RISK STRATIFICATION FOR DISEASE RECURRENCE/PERSISTENCE—AN ONGOING PROCESS

A study published by Tuttle et al.^27^ in 2010 followed patients for a median of 7 years after their original treatment and incorporated a response-to-therapy variable into the ATA risk stratification systems in order further to identify patients whose disease is likely to recur versus those likely to remain disease-free. The goal was to validate the risk-of-recurrence staging system described in the ATA guidelines and to include a “response-to-therapy after 2 years” variable in an assessment to modify the initial estimates of risk. Clinical endpoints classified patients as “no evidence of disease” or “persistent disease.” To be categorized as no evidence of disease (NED), patients had a suppressed thyroglobulin (Tg) <1 ng/mL and no structural evidence of disease. Patients with persistent disease were divided into two groups: a “biochemical incomplete” group if either suppressed or stimulated Tg was elevated but they had negative imaging, or a “structural incomplete” group if they had either positive cytology/histology, highly suspicious lymph nodes, thyroid bed nodules on ultrasound, or if findings highly suspicious for metastatic disease were seen on imaging such as radioactive iodine (RAI) scan, 18-FDG-PET scan, or other. When using the ATA classification system, 86% of patients defined as low risk remained NED, whereas 57% remained NED in the intermediate-risk and 14% NED in the high-risk groups. Not unexpectedly, the proportions were different for biochemical incomplete (11% in the low-risk group, 22% in the intermediate-risk group, and 14% in the high-risk group) and structural incomplete (3% in the low-risk group, 21% in the intermediate-risk group, and 72% in the high-risk group). Patients were re-classified into “excellent response to therapy,” “acceptable response to therapy,” or “incomplete response to therapy.” When re-classifying patients based on response to therapy, the assessed likelihood of persistent disease in the intermediate-risk group decreased from 18% to 2%, and in the high-risk group from 66% to 14%. Conversely, patients with an incomplete response to therapy had a higher likelihood of persistent/recurrent disease, and this helped identify those patients who ultimately required closer monitoring or additional therapy. In the low-risk group, addition of the response-to-therapy variable led to an increased likelihood of disease from 3% to 13%, in the intermediate-risk group from 18% to 41%, and in the high-risk group from 66% to 79%. Similar results have been seen in subsequent studies.^28^–^31^

These studies validate the ATA risk stratification system but also clearly highlighted the importance of incorporating response to therapy in the risk stratification as well as the significance of the process of risk stratification being a dynamic as opposed to static one.

The recently published 2015 ATA guidelines for management of thyroid cancer^32^ proposed additional considerations in the initial risk stratification process with an understanding that certain variables, such as those mentioned earlier, may pose a higher risk of disease recurrence/persistence and therefore should be incorporated. These guidelines demonstrate the complexity of the risk stratification process and suggest that patients fall into a continuum of risk of structural disease recurrence in patients without identifiable structural disease after initial therapy. Based on clinico-pathologic features thought to contribute to a higher or lower risk of disease recurrence, additional elements in this risk stratification process have therefore been added. These include histopathologic subtype, presence/absence as well as degree of vascular invasion, focality (unifocal versus multifocal), number and size of pathologic regional lymph nodes with metastatic disease, encapsulated versus non-encapsulated tumor, and, if known, BRAF V600E mutation status (in certain situations).

## THE ROLE OF GENETIC MUTATIONS AND MOLECULAR MARKERS IN RISK STRATIFICATION

A number of genetic mutations are known to be associated with thyroid malignancy. A recent meta-analysis^33^ of 14 studies found that patients with papillary thyroid cancer who were BRAF mutation positive had a 2.66-fold higher risk of death from their disease than those without. Patients with RAS mutation were 2.9-fold more likely to die from thyroid cancer than those without this mutation.

A retrospective multicenter analysis^34^ following patients for a median of 33 months found that the presence of BRAF mutation was associated with a statistically significant increased risk of mortality (5.3% versus 1.1%, *P*<0.001) in patients with papillary thyroid cancer. In a multivariate analysis including extra-thyroidal invasion, lymph node metastases, and distant metastases, the difference was no longer significant between those that were BRAF mutation positive or negative. Therefore, the utility of this mutation as an independent predictor of death is unclear.

The same group evaluated the value of BRAF mutation as an indicator of risk of recurrence.^35^ Over a median of 36 months, PTC recurrence was seen in 20.9% of patients who were BRAF mutation positive, versus in 11.6% of patients who were BRAF mutation negative. The hazard ratio for recurrence was 1.82 (95% CI 1.46–2.28, *P*<0.001). The difference remained statistically significant in multivariate analysis, suggesting that BRAF mutation is an independent risk factor for recurrence. To date, this is the largest cohort studied to assess the BRAF mutation as an independent variable in the risk stratification process.

A 2012 meta-analysis^36^ showed that presence of BRAF mutation in PTC was significantly associated with a higher risk of recurrence, as well as extra-thyroidal extension and lymph node metastases. As such, understanding the specific attributable risk of the BRAF mutation to recurrence is challenging. It appears that there may be an improvement in risk stratification when combining BRAF mutation status in context with other clinico-pathologic features^37^; however, additional study is needed. Not all studies have consistently shown this mutation to be associated with higher risk for a more aggressive clinical course,^38^–^40^ nor is the clinical ramification of the BRAF mutation identical in all subtypes of papillary thyroid cancer.^41^,^42^

The significance of BRAF mutation as well as other mutations and molecular markers found to be associated with an effect on clinical course, recurrence or persistence, and/or prognosis is undergoing evaluation. As we continue to learn more about the characteristics of these tumors their role in the risk stratification process is likely to gain significance, potentially even in the preoperative setting. Increasingly, nodules with indeterminate cytology on fine-needle aspiration biopsy (FNAB) are undergoing gene mutation analysis to predict the risk of malignancy.^43^–^46^

## THE ROLE OF THYROGLOBULIN IN RISK STRATIFICATION—POSTOPERATIVELY AND LONG TERM

Measurement of serum thyroglobulin (Tg) is standard in the follow-up of thyroid cancer. It is a useful tool in predicting thyroid cancer persistence, recurrence, as well as lack of disease in well-differentiated thyroid carcinoma.

In recent years, the use of postoperative or post-ablative Tg has been shown to be an important predictor of recurrence/persistence and can help guide the clinician when considering treatment and follow-up. Though the recent ATA guidelines do not incorporate Tg levels in the initial risk stratification process, Tg levels do have an intuitive role in risk stratification and can help guide the clinician in the decision-making process.

Serum Tg (suppressed or stimulated) levels at the time of ablation have been repeatedly found to correlate with Tg levels in the initial follow-up period.^47^–^50^ Therefore, they may be used in the post-surgical period to help guide treatment.

In the post-thyroidectomy pre-ablative period, in patients considered to be low risk and certain patients with intermediate risk, a Tg (suppressed or stimulated) of <1 ng/mL is suggestive of a very good prognosis.^51^ It is thought that, in these patients, treatment with radioactive iodine is likely not needed and monitoring is appropriate.^52^

The extent of surgery and completeness of resection can have an important role in a patient’s risk of persistent and/or recurrent disease. The decision regarding the initial surgical intervention and the burden of tissue/disease potentially left postoperatively warrant a separate thorough review and are not discussed here; however, a postoperative Tg level may provide the clinician with insight as to the degree of thyroid tissue remaining and may guide in the decision-making process.

More recently, it has been suggested that a postoperative stimulated Tg in combination with ultrasonography is an appropriate step in the risk stratification process after surgery for low- or intermediate-risk patients as a guiding tool in the decision on whether or not to treat with radioactive iodine.^53^

Current guidelines do not specifically outline which may be of greater or lesser significance, a stimulated or suppressed thyroglobulin, in the postoperative time frame. To date, no comparison studies have examined this period to answer this question.

As the title of this review suggests, risk stratification is an ongoing dynamic process. Following radioactive iodine treatment, patients are generally followed initially every 6–12 months with a serum Tg. In patients who are initially considered high risk, more frequent monitoring may be appropriate. Patients initially categorized as ATA low or intermediate risk, who had undergone remnant ablation/adjuvant therapy, should have a cervical ultrasound and Tg measured for evaluation of response to therapy at 6–18 months. The highest degree of sensitivity for serum Tg follows stimulation, either by thyroid hormone withdrawal or after rhTSH (recombinant human TSH) injection, and therefore a stimulated Tg may be the most useful in determining those patients who are free of disease. In those patients who achieve an excellent response to therapy (no clinical evidence of tumor, no imaging evidence of tumor, and suppressed Tg <0.2 ng/mL or stimulated Tg <1 ng/mL with negative antibodies), repeated stimulation testing may not be necessary, and the interval between subsequent Tg measurements may be lengthened to 12–24 months. Conversely, those patients who on follow-up have an indeterminate, biochemical incomplete or structural incomplete response to therapy noted on follow-up testing should be considered for more frequent monitoring, additional evaluation, and/or therapy.

## ADDITIONAL CONSIDERATIONS

Though beyond the scope of this report, the subject of using molecular markers and genetic mutations for evaluation of nodules is being investigated not only for diagnosis of malignancy but also for prognostication and for consideration as to the extent of initial therapy needed.^54^,^55^

It becomes evident that, in this increasingly common malignancy, the socioeconomic burden of disease will have a significant impact on patients’ lives as well as the health care system. As discussed above, when considering risk stratification, it is important to identify those who will remain disease-free who may not need aggressive treatment and/or follow-up. However, the paradigm continues to shift. More recently, thought has been given regarding those with known disease, to identify those who require treatment versus those in whom the risk of treatment of their malignancy may outweigh the benefit.

The recently published ATA guidelines support the concept of active surveillance for certain patients with papillary microcarcinoma. It is thought that one of the contributors to the increase in incidence of thyroid cancer is early diagnosis of thyroid cancer in clinically insignificant nodules, usually incidentally discovered nodules measuring <1 cm.^56^ In a study published by Ito et al. in 2010,^57^ it was suggested that patients diagnosed with low-risk papillary microcarcinoma may be followed with close observation and that delayed surgical treatment in the context of suspected disease progression during surveillance did not worsen outcome. A follow-up study^58^ by this group found that young age (<40) was an independent predictor of disease progression and that older patients (≥60) were less likely to demonstrate disease progression. Similarly to their previous study, however, they found that outcome may not be worsened if treatment is delayed until disease has been shown to progress. A clinically applicable method to risk-stratify those patients who may be the most appropriate candidates for active surveillance in subclinical papillary microcarcinoma has been recently proposed.^59^

The concept of active surveillance in patients with known/suspected disease has been further evaluated, with the important consideration of the clinical significance of disease versus that of additional treatment and may be an appropriate alternative in a select group of people with regional lymph node recurrence.^60^

## SUMMARY

Differentiated thyroid cancer is an increasingly common malignancy, and its prevalence is the most rapidly increasing among solid tumors. Despite the rising incidence, mortality from thyroid cancer remains low. Nevertheless, the risk of disease recurrence/persistence in the years following original treatment, though tending to decrease incrementally, is not insignificant. The societal burden is multifaceted. It is important to keep in mind that though certain disease states may be associated with an increased risk of morbidity and mortality, so too may the various treatments offered to our patients.

It has been established that though the initial staging systems may be appropriate for assessment of risk of mortality, they do not provide sufficient information to identify those patients at risk for clinically significant disease who will require more aggressive treatment and follow-up versus those who will not.

Over recent years, a number of additional variables have been identified that may aid in the initial risk stratification and guide the clinician in answering questions such as: “Is surgery necessary?”, “What is the appropriate surgery?”, and “Is radioactive iodine appropriate?”.

Finally, it is evident that risk stratification is an ongoing dynamic process and must incorporate a response-to-therapy variable. This will allow correct identification of recurrence/persistence in those patients initially thought to be low risk, and, similarly, those previously thought to be at high risk may be unencumbered by frequent unnecessary testing after displaying a successful response.

## Abbreviations:

AJCC: American Joint Committee on Cancer
ATA: American Thyroid Association
FTC: follicular thyroid cancer
NED: no evidence of disease
PTC: papillary thyroid cancer
rhTSH: recombinant human TSH
Tg: thyroglobulin
UICC: Union for International Cancer Control.

## References

[1] National Cancer Institute http://seer.cancer.gov/statfacts/html/thyro.html.

[2] National Cancer Registry-Thyroid Cancer Incidence Tables. Ministry of Health Israel. http://bit.ly/1OVBIDw. Accessed September 25, 2015 [Hebrew].

[3] Edge SB, Byrd DR, Compton CC, Fritz AG, Greene FL, Trotti A (2010). AJCC Cancer Staging Handbook.

[4] Byar DP, Green SB, Dor P (1979). A prognostic index for thyroid carcinoma: a study of the EORTC Thyroid Cancer Cooperative Group. Eur J Cancer.

[5] Hay ID, Grant CS, Taylor WF, McConahey WM (1987). Ipsilateral lobectomy versus bilateral lobar resection in papillary thyroid carcinoma: a retrospective analysis of surgical outcome using a novel prognostic scoring system. Surgery.

[6] Cady B, Rossi R (1988). An expanded view of risk-group definition in differentiated thyroid carcinoma. Surgery.

[7] Hay ID, Bergstralh EJ, Goellner JR, Ebersold JR, Grant CS (1993). Predicting outcome in papillary thyroid carcinoma: development of a reliable prognostic scoring system in a cohort of 1779 patients surgically treated at one institution during 1940 through 1989. Surgery.

[8] Shaha AR, Loree TR, Shah JP (1994). Intermediate-risk group for differentiated carcinoma of the thyroid. Surgery.

[9] Lang BH, Lo CY, Chan WF, Lam KY, Wan KY (2007). Staging systems for papillary thyroid carcinoma: a review and comparison. Ann Surg.

[10] Lang BH, Lo CY, Chan WF, Lam KY, Wan KY (2007). Staging systems for follicular thyroid carcinoma: application to 171 consecutive patients treated in a tertiary referral centre. Endocr Relat Cancer.

[11] D’Avanso A, Ituarte P, Treseler P (2004). Prognostic scoring systems in patients with follicular thyroid cancer: a comparison of different staging systems in predicting the patient outcome. Thyroid.

[12] DeLellis RA, Lloyd RD, Heitz PU, Eng C (2004). WHO: Pathology and Genetics Tumours of Endocrine Organs.

[13] Siegel R, DeSantis C, Virgo K (2012). Cancer treatment and survivorship statistics, 2012. CA Cancer J Clin.

[14] van den Brekel MW, Hekkenberg RJ, Asa SL, Tomlinson G, Rosen IB, Freeman JL (1997). Prognostic features in tall cell papillary carcinoma and insular thyroid carcinoma. Laryngoscope.

[15] Yamashita H, Noguchi S, Murakami N, Kawamoto H, Watanabe S (1997). Extracapsular invasion of lymph node metastasis is an indicator of distant metastasis and poor prognosis in patients with thyroid papillary carcinoma. Cancer.

[16] Randolph GW, Duh QY, Heller KS (2012). The prognostic significance of nodal metastases from papillary thyroid carcinoma can be stratified based on the size and number of metastatic lymph nodes, as well as the presence of extranodal extension. Thyroid.

[17] Gardner RE, Tuttle RM, Burman KD (2000). Prognostic importance of vascular invasion in papillary thyroid carcinoma. Arch Otolaryngol Head Neck Surg.

[18] Falvo L, Catania A, D’Andrea V, Marzullo A, Giustiniani MC, De Antoni E (2005). Prognostic importance of histologic vascular invasion in papillary thyroid carcinoma. Ann Surg.

[19] Rivera M, Ricarte-Fiho J, Knauf J (2010). Molecular genotyping of papillary thyroid carcinoma follicular variant according to its histological subtypes (encapsulated vs infiltrative) reveals distinct BRAF and RAS mutation patterns. Mod Pathol.

[20] Piana S, Frasoldat A, Di Felice E, Gardini G, Tallini G, Rosai J (2010). Encapsulated well-differentiated follicular patterned thyroid carcinomas do not play a significant role in the fatality rates from thyroid carcinoma. Am J Surg Pathol.

[21] Proietti A, Giannini R, Ugolini C (2010). BRAF status of follicular variant of papillary thyroid carcinoma and its relationship to its clinical and cytological features. Thyroid.

[22] Tuttle RM (2008). Risk-adapted management of thyroid cancer. Endocr Pract.

[23] Tuttle RM, Leboeuf R, Shaha AR (2008). Medical management of thyroid cancer: a risk adapted approach. J Surg Oncol.

[24] Watkinson JC, British Thyroid Association (2004). The British Thyroid Association guidelines for the management of thyroid cancer in adults. Nucl Med Commun.

[25] Pacini F, Schlumberger M, Dralle H (2006). European consensus for the management of patients with differentiated thyroid carcinoma of the follicular epithelium. Eur J Endocrinol.

[26] Cooper DS, Doherty GM, American Thyroid Association (ATA) Guidelines Taskforce on Thyroid Nodules and Differentiated Thyroid Cancer (2009). Revised American Thyroid Association management guidelines for patients with thyroid nodules and differentiated thyroid cancer. Thyroid.

[27] Tuttle RM, Tala H, Shah J (2010). Estimating risk of recurrence in differentiated thyroid cancer after total thyroidectomy and radioactive iodine remnant ablation: using response to therapy variables to modify the initial risk estimates predicted by the new American Thyroid Association staging system. Thyroid.

[28] Vaisman F, Momesso D, Bulzico DA (2012). Spontaneous remission in thyroid cancer patients after biochemical incomplete response to initial therapy. Clin Endocrinol (Oxf).

[29] Castagna MG, Maino F, Cipri C (2011). Delayed risk stratification, to include the response to initial treatment (surgery and radioiodine ablation), has better outcome productivity in differentiated thyroid cancer patients. Eur J Endocrinol.

[30] Pitoia F, Bueno F, Urciuoli C, Abelleira E, Cross G, Tuttle RM (2013). Outcomes of patients with differentiated thyroid cancer risk-stratified according to the American Thyroid Association and Latin American Thyroid Society risk of recurrence classification systems. Thyroid.

[31] Hong CM, Lee WK, Jeong SY, Lee SW, Ahn BC, Lee J (2014). Superiority of delayed risk stratification in differentiated thyroid cancer after total thyroidectomy and radioactive iodine ablation. Nucl Med Commun.

[32] Haugen BR, Alexander EK, Bible KC (2015). American Thyroid Association management guidelines for adult patients with thyroid nodules and differentiated thyroid cancer. Thyroid.

[33] Pak K, Suh S, Kim SJ, Kim IJ (2015). Prognostic value of genetic mutations in thyroid cancer: a meta-analysis. Thyroid.

[34] Xing M, Alzahrani AS, Carson KA (2013). Association between BRAF V600E mutation and mortality in patients with papillary thyroid cancer. JAMA.

[35] Xing M, Alzahrani AS, Carson KA (2015). Association between BRAF V600E mutation and recurrence of papillary thyroid cancer. J Clin Oncol.

[36] Tufano RP, Teixeira GV, Bishop J, Carson KA, Xing M (2012). BRAF mutation in papillary thyroid cancer and its value in tailoring initial treatment: a systematic review and meta-analysis. Medicine (Baltimore).

[37] Prescott JD, Sadow PM, Hodin RA (2012). BRAF V600E status adds incremental value to current risk classification systems in predicting papillary thyroid carcinoma recurrence. Surgery.

[38] Sancisi V, Nicoli D, Ragazzi M, Piana S, Ciarrocchi A (2012). BRAFV600E mutation does not mean distant metastasis in thyroid papillary carcinoma. J Clin Endocrinol Metab.

[39] Barbaro D, Incensati RM, Materazzi G (2014). The BRAF V600E mutation in papillary thyroid cancer with positive or suspected pre-surgical cytological finding is not associated with advanced stages or worse prognosis. Endocrine.

[40] Gouveia C, Can NT, Bostrom A, Grenert JP, van Zante A, Orloff LA (2013). Lack of association of BRAF mutation with negative prognostic indicators in papillary thyroid carcinoma: the University of California, San Francisco experience. JAMA Otolaryngol Head Neck Surg.

[41] Tallini G, de Biase D, Durante C (2015). BRAF V600E and risk stratification of thyroid microcarcinoma: a multicenter pathological and clinical study. Mod Pathol.

[42] Park YJ, Kim YA, Lee YJ (2010). Papillary microcarcinoma in comparison with larger papillary thyroid carcinoma in BRAF (V600E) mutation, clinico-pathological features, and immunohistochemical findings. Head Neck.

[43] Nikiforov YE, Carty SE, Chiosea SI (2015). Impact of the multi-gene ThyroSeq next-generation sequencing assay on cancer diagnosis in thyroid nodules with atypia of undetermined significance/follicular lesion of undetermined significance cytology. Thyroid.

[44] Nikiforov YE, Carty SE, Chiosea SI (2014). Highly accurate diagnosis of cancer in thyroid nodules with follicular neoplasm/suspicious for a follicular neoplasm cytology by ThyroSeq v2 next-generation sequencing assay. Cancer.

[45] Alexander EK, Kennedy GC, Baloch ZW (2012). Preoperative diagnosis of benign thyroid nodules with indeterminate cytology. N Engl J Med.

[46] Kloos RT, Reynolds JD, Walsh PS (2013). Does addition of BRAF V600E mutation testing modify sensitivity or specificity of the Afirma Gene Expression Classifier in cytologically indeterminate thyroid nodules?. J Clin Endocrinol Metab.

[47] Kim TY, Kim WB, Kim ES (2005). Serum thyroglobulin levels at the time of 131I remnant ablation just after thyroidectomy are useful for early prediction of clinical recurrence in low-risk patients with differentiated thyroid carcinoma. J Clin Endocrinol Metab.

[48] Giovanella L, Ceriani L, Ghelfo A, Keller F (2005). Thyroglobulin assay 4 weeks after thyroidectomy predicts outcome in low-risk papillary thyroid carcinoma. Clin Chem Lab Med.

[49] Piccardo A, Arecco F, Morbelli S (2010). Low thyroglobulin concentrations after thyroidectomy increase the prognostic value of undetectable thyroglobulin levels on levo-thyroxine suppressive treatment in low-risk differentiated thyroid cancer. J Endocrinol Invest.

[50] Polachek A, Hirsch D, Tzvetov G (2011). Prognostic value of post-thyroidectomy thyroglobulin levels in patients with differentiated thyroid cancer. J Endocrinol Invest.

[51] Ibrahimpasic T, Nixon IJ, Palmer FL (2012). Undetectable thyroglobulin after total thyroidectomy in patients with low- and intermediate-risk papillary thyroid cancer--is there a need for radioactive iodine therapy?. Surgery.

[52] Vaisman A, Orlov S, Yip J (2010). Application of post-surgical stimulated thyroglobulin for radioiodine remnant ablation selection in low-risk papillary thyroid carcinoma. Head Neck.

[53] Orlov S, Salari F, Kashat L (2015). Post-operative stimulated thyroglobulin and neck ultrasound as personalized criteria for risk stratification and radioactive iodine selection in low- and intermediate-risk papillary thyroid cancer. Endocrine.

[54] Shrestha RT, Karunamurthy A, Amin K, Nikiforov YE, Caramori ML (2015). multiple mutations detected preoperatively may predict aggressive behavior of papillary thyroid cancer and guide management - a case report. Thyroid.

[55] An JH, Song KH, Kim SK (2015). RAS mutations in indeterminate thyroid nodules are predictive of the follicular variant of papillary thyroid carcinoma. Clin Endocrinol (Oxf).

[56] Davies L, Welch HG (2014). Current thyroid cancer trends in the United States. JAMA Otolaryngol Head Neck Surg.

[57] Ito Y, Miyauchi A, Inoue H (2010). An observational trial for papillary thyroid microcarcinoma in Japanese patients. World J Surg.

[58] Ito Y, Miyauchi A, Kihara M, Higashiyama T, Kobayashi K, Miya A (2014). Patient age is significantly related to the progression of papillary micro-carcinoma of the thyroid under observation. Thyroid.

[59] Brito JP, Ito Y, Miyauchi A, Tuttle RM (2016). A clinical framework to facilitate risk stratification when considering an active surveillance alternative to immediate biopsy and surgery in papillary micro-carcinoma. Thyroid.

[60] Robenshtok E, Fish S, Bach A, Domínguez JM, Shaha A, Tuttle RM (2012). Suspicious cervical lymph nodes detected after thyroidectomy for papillary thyroid cancer usually remain stable over years in properly selected patients. J Clin Endocrinol Metab.

